# Role of duct excision surgery in the treatment of pathological nipple discharge and detection of breast carcinoma: systematic review

**DOI:** 10.1093/bjsopen/zrad066

**Published:** 2023-07-17

**Authors:** Seher Makineli, Jan Willem M van Wijnbergen, Menno R Vriens, Paul J van Diest, Arjen J Witkamp

**Affiliations:** Department of Surgical Oncology, University Medical Center, Utrecht, The Netherlands; Department of Surgical Oncology, University Medical Center, Utrecht, The Netherlands; Department of Surgical Oncology, University Medical Center, Utrecht, The Netherlands; Department of Pathology, University Medical Center, Utrecht, The Netherlands; Department of Surgical Oncology, University Medical Center, Utrecht, The Netherlands

## Abstract

**Background:**

The role of duct excision surgery is not clearly defined in patients with pathological nipple discharge without other clinical and radiological abnormalities. The primary aim of this systematic review was to determine the malignancy rate in patients with pathological nipple discharge after duct excision surgery (microdochectomy/major duct excision). The secondary aims were to determine the recurrence rate of pathological nipple discharge after surgery and to assess breast cancer development after surgery.

**Methods:**

MEDLINE and Embase were searched from inception to March 2023, using search terms related to ‘nipple discharge’, ‘nipple fluid’, ‘microdochectomy’, ‘duct excision’, and ‘minimally invasive surgical procedure’. Studies reporting data about women who underwent duct excision surgery for pathological nipple discharge without clinical and radiological suspicion of breast cancer, as well as reporting data on women diagnosed with breast cancer after duct excision surgery, were included.

**Results:**

A total of 318 titles were identified, of which nine publications were included in the analysis. This resulted in 1108 patients with pathological nipple discharge who underwent a duct excision. The weighted mean rate of malignancy after duct excision surgery was 8.1 per cent (ranging from 2.3 to 13.5 per cent). Three studies described the recurrence rate of pathological nipple discharge (ranging from 0 to 12 per cent) and two studies reported breast cancer development in the follow-up in a total of three patients (less than 1 per cent).

**Conclusion:**

The malignancy rate after duct excision surgery for pathological nipple discharge was low in patients with pathological nipple discharge without radiological and clinical abnormalities and approximately 9 of 10 patients undergo surgery for a benign cause. Improvement of the diagnostic and therapeutic workup is needed to prevent patients from undergoing (unnecessary) exploratory surgery.

## Teaser

In patients with pathological nipple discharge without radiological and clinical abnormalities, the malignancy rate after duct excision surgery was 8.1 per cent. The recurrence rate of pathological nipple discharge after surgery ranged from 0 to 12 per cent. Improvement of the diagnostic and therapeutic workup is needed to prevent patients from undergoing exploratory surgery.

## Introduction

Nipple discharge is a common symptom, reported in 2–5 per cent of all women and in 8 per cent of women presenting with a breast complaint^1–3^. When nipple discharge is unilateral, spontaneous, bloody or serous, and arising from a single duct orifice of the nipple, it is defined as pathological nipple discharge (PND)^4^. The common causes of PND are benign (ductal ectasia and intraductal papillomas)^5,6^. However, it is also associated with breast cancer^7,8^.

PND is a clinical diagnosis confirmed through patient history and physical examination. In patients with confirmed PND, current guidelines advise further evaluation with mammography and breast ultrasound to rule out underlying malignancy. These techniques both have a low sensitivity (22 and 50 per cent respectively) in detecting malignancy when PND is the only complaint^9,10^. On the other hand, MRI has a high sensitivity in detecting malignancy at the cost of low specificity^10^. Furthermore, detecting small lesions with MRI has proven to be difficult^11,12^. Hence, surgical excision and histopathological examination are needed to confirm diagnoses made with MRI^13,14^. Therefore, MRI is of limited added value to patients with PND. Another available diagnostic technique is ductoscopy, which has not yet been widely adopted, despite advances and increasing interest in recent decades^15–17^.

In patients suffering from PND without radiological and clinical abnormalities, surgical excision is traditionally required to rule out malignancy. Two widely adopted techniques of surgical excision are microdochectomy and major duct excision. Microdochectomy is the excision of a single duct, and major duct excision is the removal of all lactiferous ducts under the nipple. These procedures are performed ‘blindly’ and carry risks. Adverse cosmetic outcomes, as well as altered lactation and sensitivity of the nipple, have been reported^7,18,19^. Previous studies reported a malignancy rate of 9.3–37 per cent in patients suffering from PND, but these studies also included patients with radiological and/or clinical suspicion (palpable mass) of malignancy^19–22^. Therefore, the malignancy rate of patients suffering from PND without radiological and clinical suspicion is not yet accurately represented because the data are sparse and studied within small populations. Moreover, the wide range of malignancy rates in previous studies makes interpretation of data difficult. A more representative malignancy rate in this population could be relevant to help better identify patients at risk and potentially prevent unnecessary exploratory surgery.

Besides its diagnostic value, duct excision surgery is also thought to have a therapeutic effect on PND complaints. Currently, there is no overview of the therapeutic effect and the recurrence rate of PND after surgery. Also, there are a lack of data about breast cancer development after duct excision surgery in patients suffering from PND.

The aim of this systematic review was to assess the rate of malignancy in patients with PND undergoing duct excision surgery, the recurrence rate of PND after surgery, and the development of breast carcinoma in the follow-up after surgery for PND in patients without other clinical or radiological abnormalities.

## Methods

This systematic review was designed and reported according to the principles of the PRISMA 2020 guidelines for reporting systematic reviews^23^. A checklist is presented in the *Supplementary material*. The research was registered in the International Prospective Register of Systematic Reviews (PROSPERO 2022 CRD42022306622)^24^.

### Data sources and searches

With the help of an experienced librarian, a broad electronic search was conducted using index terms and free-text words in MEDLINE and Embase from inception to March 2023, without language restrictions. Scopus was used to fine-tune the initial MEDLINE search. Also, forward citation analyses and backward bibliographic sampling of included articles were conducted. This search strategy included terms related to ‘nipple discharge’, ‘nipple fluid’, ‘microdochectomy’, ‘duct excision’, and ‘minimally invasive surgical procedure’. The full search is shown in *Table S1*. Reference lists from eligible articles were also examined to identify publications. The last search was conducted on 9 March 2023.

### Study selection

Citations from all search results were downloaded and merged using Rayyan, an online program for systematic reviews^25^. According to the predefined inclusion and exclusion criteria, two authors (S.M. and J.W.M.v.W.) screened titles and abstracts independently. Then, full-text articles were reviewed for eligibility independently by the same authors. Disagreements were settled by consensus or a third author (A.J.W.) was consulted for adjudication.

Studies were included that reported data about women with PND without clinical and radiological suspicion of breast cancer and also reported data about women who were diagnosed with breast cancer after duct excision surgery. PND was defined as spontaneous, single-duct nipple discharge during a non-lactational interval, persisting for more than 3 months. This review used the definition of malignancy as described in the included studies.

Imaging criteria were the absence of abnormalities on radiological examination (lesions suspected of being indicative of breast cancer) and the use of diagnostic mammography and ultrasound in the workup of PND, with or without biopsy.

Studies were excluded if they had any of the following characteristics: abstract-only publications, case reports, case series, papers from which the full-text was missing or unavailable, and conference abstracts; insufficient data or irrelevant research question for this review; studies written in languages other than English, Dutch, Turkish, German, or Spanish; use of interventional ductoscopy before intervention; data from patients with a palpable mass; and studies with a study population before 1995 because of discordant diagnostic possibilities compared with the current diagnostic workup.

### Data extraction and analysis

The data extraction was performed independently by two authors (S.M. and J.W.M.v.W.), using an Excel-based spreadsheet (Microsoft^®^). Outcomes reported in any article are summarized qualitatively in this systematic review. These include information on publication details, study design, number of eligible patients in each study, complication rate, follow-up, recurrence of complaints, and histological analysis. All data were tabulated and presented as percentages. A modified rating grade (from 1 to 5) from the Oxford Centre for Evidence-Based Medicine was used to determine the quality of the evidence^26^. Re-excision surgery for malignancy was not noted as a complication.

## Results

### Search outcome and study characteristics

**Fig. 1:**
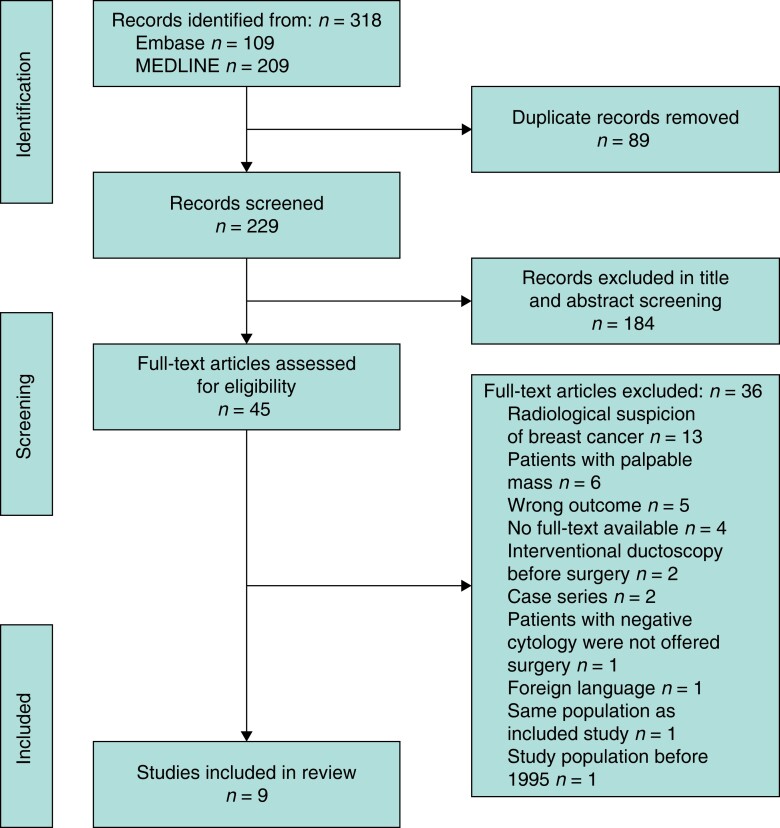
PRISMA flow diagram of literature search and selection of studies

The study selection process with reasons for exclusion is described in *Fig. 1*. The literature search resulted in 209 articles in MEDLINE and 109 articles in Embase, giving a total of 318 articles. After removing duplicate publications, 229 titles and abstracts were screened for eligibility, of which 184 articles were excluded for not meeting the inclusion criteria. Full-text articles were reviewed for the 45 studies identified as potentially eligible by one or both reviewers. Nine articles were selected for inclusion in the final analysis.

The study characteristics and results are summarized in *Table 1*. Of the selected nine studies, six were from Europe, two were from North America, and one was from Asia. The sample size of these studies ranged from 33 to 214, resulting in a total of 1108 patients included in this study. The age of patients ranged from 17 to 88 years (the median age ranged from 47.8 to 56.7 years). The quality rating score for the level of evidence ranged from 1 to 3.

**Table 1:** Study characteristics and results

Study	Country	Study design	Quality rating score*	Sample size	Pathology: benign† (%)	Pathology: malignant (%)	Follow-up (years)	Follow-up: breast cancer development	Recurrence of PND (%)
Çetin and Sıkar^27^ 2020	Turkey	Retrospective cohort	3	111	86.5	DCIS: 7.2; IC: 6.3	–	–	0
Foulkes *et al*.^28^ 2011	UK	Prospective cohort	2	194	94	DCIS: 4; IC: 2	–	–	12
Gui *et al*.^29^ 2018	UK	Randomized clinical trial	1	66	92.4	DCIS: 7.6; IC: 0	3–9	No	3
Hahn *et al*.^30^ 2009	Germany	Prospective cohort	3	33	93.9	DCIS: 3; IC: 3	0.5	–	–
Lustig *et al*.^31^ 2019	Canada	Retrospective cohort	3	155	87	DCIS: 10; IC: 3	–	–	–
Ohlinger *et al*.^32^ 2020	Germany	Prospective cohort	2	214	94.9	DCIS: 4.6; IC: 0.5	–	–	–
Richards *et al*.^33^ 2007	UK	Retrospective cohort	3	86	97.7	DCIS: 1.2; LCIS: 1.1; IC: 0	–	–	–
Simpson *et al*.^34^ 2009	Canada	Retrospective cohort	3	65	95.4	DCIS: 3.1; IC: 1.5	–	–	–
Wong Chung *et al*.^35^ 2016	The Netherlands	Retrospective cohort	3	184	89.2	DCIS: 7.6; IC: 3.3	3–13	Yes, in three patients	–

*Quality rating score for studies and evidence. 1: Properly randomized clinical trial; systematic review with meta-analysis. 2: Well-designed controlled trial without randomization; prospective comparative cohort trial. 3: Case–control studies; retrospective cohort study. 4: Case series with or without intervention; cross-sectional study. 5: Opinion of respected authorities; case reports. †Benign tissue, intraductal papilloma, hyperplasia, duct ectasia, atypia, inflammatory, fibroadenoma, fibrocystic changes, and sclerosing lesions. PND, pathological nipple discharge; DCIS, ductal carcinoma *in situ*; IC, invasive carcinoma; LCIS, lobular carcinoma *in situ*; –, missing data.

### Histopathological findings

**Fig. 2:**
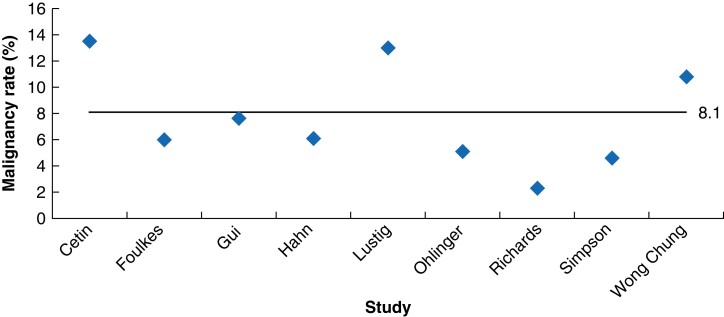
Malignancy rates after duct excision surgery for pathological nipple discharge in the included studies The histopathological results of malignancy after duct excision surgery in patients with pathological nipple discharge without radiological and clinical abnormalities from all included studies. The weighted average rate of malignancy was 8.1 per cent.

The majority of the lesions at microdochectomy and major duct excision were benign (ranging from 86.5 to 97.7 per cent). Reported benign lesions were unspecified benign tissue, intraductal papilloma, hyperplasia, duct ectasia, atypia, inflammatory, fibroadenoma, fibrocystic changes, and sclerosing lesions. Reported malignant lesions were ductal carcinoma *in situ* (DCIS), invasive carcinoma, and, in one study, lobular carcinoma *in situ* (LCIS). DCIS was the most common malignant lesion in the study population. Based on the selected studies, the weighted average rate of malignancy after duct excision surgery was 8.1 per cent (ranging from 2.3 to 13.5 per cent), as shown in *Fig. 2*.

### Follow-up, breast cancer development, and recurrence of pathological nipple discharge

The length of follow-up after duct excision surgery was noted in three studies (ranging from 0.5 to 13 years) (*Table 1*). The other six studies did not report follow-up data. Two of the studies reported on the development of breast cancer; Gui *et al*.^29^ reported no breast cancer development during a follow-up of 3–9 years (zero of 66 patients) and Wong Chung *et al*.^35^ reported three patients with breast cancer during a follow-up interval of 3–12 years (three of 184 patients; 1.6 per cent). These three patients had primary benign histology after surgery and developed a tumour in the ipsilateral breast, but at different locations. Those malignancies were considered as new and not related to the initial duct excision surgery^35^.

Three studies described the recurrence rate of PND (ranging from 0 to 12 per cent). Çetin *et al*.^27^ reported a recurrence rate of 0 per cent; however, the follow-up interval was not reported.

### Complications

Complications were reported in 15 patients of the total study population of 1108 patients (1.4 per cent) after duct excision surgery (*Table S2*): five patients had haematomas, five patients had postoperative surgical site infections, four patients had postoperative seromas, and one patient had partial necrosis of the areola, which healed with conservative treatment. The complication rate in the included studies ranged from 0 to 9 per cent; three studies did not report complication rates.

## Discussion

Here, the role of duct excision surgery in the detection of breast carcinoma in patients with PND is reported. Breast carcinoma was found in only 8.1 per cent of patients with PND without radiological and clinical abnormalities. This means that the majority of patients underwent surgery for benign lesions in this study. Thus, improvement of the diagnostic and therapeutic workup is needed to prevent patients from undergoing unnecessary exploratory surgery. Furthermore, recurrence of PND after duct excision surgery was reported in 0–12 per cent of patients, meaning that excision surgery cures PND complaints in more than 88 per cent of patients. Breast cancer development was poorly described in the included studies and could not be appropriately assessed.

Previous studies examining duct excision surgery did not differentiate between studies reporting cases with or without clinical abnormalities (palpable mass) and/or radiological abnormalities. These studies with clinical and/or radiological abnormalities reported a relatively high malignancy rate, ranging from 9.3 to 37 per cent^5,19–21,36^, compared with the malignancy rate in this review. This difference is attributed to the presence of a palpable mass and radiological abnormalities and the chance of finding malignancy upon histopathological examination^37–39^.

The complications reported after surgery were concordant with previous results^7,18,19^. According to the authors’ study population, duct excision surgery is a safe intervention, with a low complication rate of 1.4 per cent in the total study population. Nevertheless, it is performed under general anaesthesia, and re-excision is needed in patients with histopathological confirmation of malignancy. Therefore, opting for surgical excision should be carefully considered.

Nowadays, in the standard workup of PND in women above 40 years, mammography and ultrasound are the key diagnostic methods to rule out malignancy^14^. Studies that included patients before 1995 were excluded in this review because of discordant diagnostic possibilities compared with the current diagnostic workup. Therefore, the largest report on microdochectomy for PND, which showed a malignancy rate of 23.9 per cent in 915 patients, was excluded because no ultrasound was performed before microdochectomy^7^. Studies were also excluded when interventional ductoscopy was performed before surgical intervention. According to previous studies, the therapeutic value of ductoscopy makes it possible to remove intraductal lesions^15,40^. Also, according to a previous study by the authors, ductoscopy prevented surgery in two of three patients with PND^17^. These findings would distort the results of this review and could lead to a higher malignancy rate due to a preselection bias of the population.

Furthermore, there was a wide range of ages for the included patients. The effect of age is not clear regarding the decision for surgical treatment. According to one included study, the median age of patients with bloodstained discharge due to breast cancer was higher than that of the patients with benign disease. Moreover, it has been suggested that a conservative policy could be adopted for women under the age of 40 years^41^. According to this review, this statement cannot be supported because of a lack of information about the ages of the included patients. Yet, this information could be crucial in the decision for the workup of patients with PND and needs further evaluation.

The role of surgery in the actual treatment of PND was described in just three studies that reported a recurrence rate of PND ranging from 0 to 12 per cent^27–29^. Gui *et al*.^29^ reported a follow-up interval of 3–9 years; in the other two studies, the follow-up interval was not specified. These results show that duct excision surgery cures PND in more than 88 per cent of patients. This was in line with other studies; Chang *et al*.^42^ reported no recurrence, and Dillon *et al*.^8^ reported 9 per cent recurrence of PND in a median interval of 7 months.

Furthermore, the included studies poorly described breast cancer development in patients after duct excision surgery. One study reported no breast cancer development during a follow-up of 3–9 years^29^ and, in one study, three patients developed breast cancer during a follow-up of 3–12 years^35^. These three patients had a tumour in the ipsilateral breast, but at different locations and after such a time interval that Wong Chung *et al*.^35^ considered these as ‘*de novo*’ malignancies. Nevertheless, these malignancies may have developed after a false-negative microdochectomy. Dillon *et al*.^8^ reported three patients diagnosed with malignancy at 2, 8, and 9 years after the initial resection. However, it was not clear if these malignancies were ‘*de novo*’ or a result of the initial complaints and operation. More research is needed about breast cancer development during follow-up after duct excision surgery to address the efficacy of the procedure and help in decision-making in the workup of patients with PND.

To prevent patients from undergoing unnecessary surgery, the focus should be on improving the diagnostic and therapeutic capabilities for intraductal breast lesions. Nowadays, a percutaneous core or vacuum-assisted biopsy is performed to obtain tissue for histopathological examination. However, when the diagnosis after biopsy remains unclear, or a benign intraductal lesion such as a papilloma is found and the nipple discharge persists after biopsy, a duct excision surgery is recommended. An additional diagnostic and interventional procedure such as ductoscopy in the workup of PND without clinical or radiological abnormalities can improve the selection of patients for surgical procedures, as it is possible to remove intraductal lesions and treat PND ductoscopically. When no or histologically proven benign lesions are found during ductoscopy, surgical procedures such as a (major) duct excision have, in the authors’ opinion, no additional diagnostic value.

This review has limitations resulting from the quality and scope of articles identified through the systematic review. There is only a limited number of studies investigating the development of breast cancer in patients with PND after duct excision surgery. Also, few studies describe the therapeutic effect of duct excision surgery and the recurrence of complaints. Due to the heterogeneous designs of the included studies and limited data, it was not possible to perform a meta-analysis, which limited the strength of the evidence. In addition, in most included articles, the findings on imaging were not defined using the breast imaging-reporting and data system (BI-RADS) classification, which is a widely accepted reporting system for imaging of the breast and applies to mammography, ultrasound, and MRI. Therefore, papers were only included when the imaging criteria were not suspicious of breast cancer and the duct excision surgery was performed because the nature of the discharge caused concern. The wide range of radiological abnormalities and differing inclusion criteria for patients represent limitations. Thus, further prospective research is required to follow-up patients with PND after duct excision surgery to generate accurate data about recurrence of PND and breast cancer development.

## Supplementary Material

zrad066_Supplementary_Data

## Data Availability

Data are available by request to the corresponding author.

## References

[1] Dupont SC , Boughey JC, Jimenez RE, Hoskin TL, Hieken TJ. Frequency of diagnosis of cancer or high-risk lesion at operation for pathologic nipple discharge. Surgery 2015;158:988–995 26243343 10.1016/j.surg.2015.05.020

[2] Gulay H , Bora S, Kilicturgay S, Hamaloglu E, Goksel H. Management of nipple discharge. J Am Coll Surg 1994;178:471–474 8167884

[3] Leis HP Jr , Greene FL, Cammarata A, Hilfer SE. Nipple discharge: surgical significance. South Med J 1988;81:20–26 3336795 10.1097/00007611-198801000-00005

[4] Onstad M , Stuckey A. Benign breast disorders. Obstet Gynecol Clin North Am 2013;40:459–473 24021252 10.1016/j.ogc.2013.05.004

[5] Goksel HA , Yagmurdur MC, Demirhan B, Isıklar I, Karakayali H, Bilgin N et al Management strategies for patients with nipple discharge. Langenbecks Arch Surg 2005;390:52–58 15372239 10.1007/s00423-004-0515-6

[6] King T , Carter K, Bolton J, Fuhrman G. A simple approach to nipple discharge. Am Surg 2000;66:960–966 11261625

[7] Montroni I , Santini D, Zucchini G, Fiacchi M, Zanotti S, Ugolini G et al Nipple discharge: is its significance as a risk factor for breast cancer fully understood? Observational study including 915 consecutive patients who underwent selective duct excision. Breast Cancer Res Treat 2010;123:895–900 20354781 10.1007/s10549-010-0815-1

[8] Dillon MF , Mohd Nazri SR, Nasir S, McDermott EW, Evoy D, Crotty TB et al The role of major duct excision and microdochectomy in the detection of breast carcinoma. BMC Cancer 2006;6:164 16796740 10.1186/1471-2407-6-164 PMC1539014

[9] Bahl M , Baker JA, Greenup RA, Ghate SV. Diagnostic value of ultrasound in female patients with nipple discharge. Am J Roentgenol 2015;205:203–208 26102400 10.2214/AJR.14.13354

[10] Filipe MD , Patuleia SIS, de Jong VMT, Vriens MR, van Diest PJ, Witkamp AJ. Network meta-analysis for the diagnostic approach to pathologic nipple discharge. Clin Breast Cancer 2020;20:e723–e748 32665191 10.1016/j.clbc.2020.05.015

[11] Sanders LM , Daigle M. The rightful role of MRI after negative conventional imaging in the management of bloody nipple discharge. Breast J 2016;22:209–212 26684050 10.1111/tbj.12551

[12] van Gelder L , Bisschops RHC, Menke-Pluymers MBE, Westenend PJ, Plaisier PW. Magnetic resonance imaging in patients with unilateral bloody nipple discharge; useful when conventional diagnostics are negative? World J Surg 2015;39:184–186 25123174 10.1007/s00268-014-2701-1

[13] de Paula IB , Campos AM. Breast imaging in patients with nipple discharge. Radiol Bras 2017;50:383–388 29307929 10.1590/0100-3984.2016.0103 PMC5746883

[14] Lee SJ , Trikha S, Moy L, Baron P, diFlorio RM, Green ED et al ACR Appropriateness Criteria^®^ evaluation of nipple discharge. J Am Coll Radiol 2017;14:S138–S153 28473070 10.1016/j.jacr.2017.01.030

[15] Waaijer L , van Diest PJ, Verkooijen HM, Dijkstra NE, van der Pol CC, Borel Rinkes IHM et al Interventional ductoscopy in patients with pathological nipple discharge. Br J Surg 2015;102:1639–1648 26447629 10.1002/bjs.9950

[16] Kamali S , Bender O, Kamali GH, Aydin MT, Karatepe O, Yuney E. Diagnostic and therapeutic value of ductoscopy in nipple discharge and intraductal proliferations compared with standard methods. Breast Cancer 2014;21:154–161 22669683 10.1007/s12282-012-0377-7

[17] Filipe MD , Waaijer L, van der Pol C, van Diest PJ, Witkamp AJ. Interventional ductoscopy as an alternative for major duct excision or microdochectomy in women suffering pathologic nipple discharge: a single-center experience. Clin Breast Cancer 2020;20:e334–e343 32081573 10.1016/j.clbc.2019.12.008

[18] Sakorafas GH . Nipple discharge: current diagnostic and therapeutic approaches. Cancer Treat Rev 2001;27:275–282 11871863 10.1053/ctrv.2001.0234

[19] Morrogh M , Park A, Elkin EB, King TA. Lessons learned from 416 cases of nipple discharge of the breast. Am J Surg 2010;200:73–80 20079481 10.1016/j.amjsurg.2009.06.021

[20] Lau S , Küchenmeister I, Stachs A, Gerber B, Krause A, Reimer T. Pathologic nipple discharge: surgery is imperative in postmenopausal women. Ann Surg Oncol 2005;12:546–551 15889216 10.1245/ASO.2005.04.013

[21] Jin L , Zhu L, Li S, Zeng Y, Haixiong L, Su F et al Predictors of malignancy for female patients with suspicious nipple discharge: a retrospective study. Anticancer Res 2017;37:4655–4658 28739767 10.21873/anticanres.11868

[22] Li GZ , Wong SM, Lester S, Nakhlis F. Evaluating the risk of underlying malignancy in patients with pathologic nipple discharge. Breast J 2018;24:624–627 29520933 10.1111/tbj.13018

[23] Page MJ , McKenzie JE, Bossuyt PM, Boutron I, Hoffmann TC, Mulrow CD et al The PRISMA 2020 statement: an updated guideline for reporting systematic reviews. Syst Rev 2021;10:89 33781348 10.1186/s13643-021-01626-4 PMC8008539

[24] Sideri S , Papageorgiou SN, Eliades T. Registration in the international prospective register of systematic reviews (PROSPERO) of systematic review protocols was associated with increased review quality. J Clin Epidemiol 2018;100:103–110 29339215 10.1016/j.jclinepi.2018.01.003

[25] *Rayyan—AI Powered Tool for Systematic Literature Reviews* . 2021. https://www.rayyan.ai/ (accessed 9 March 2023)

[26] Oxford Centre for Evidence-Based Medicine . *Levels of Evidence (March 2009)*. https://www.cebm.ox.ac.uk/resources/levels-of-evidence/oxford-centre-for-evidence-based-medicine-levels-of-evidence-march-2009 (accessed 5 June 2022)

[27] Çetin K , Sıkar HE. Evaluation and management of pathological nipple discharges without using intraductal imaging methods. Ir J Med Sci 2020;189:451–460 31631245 10.1007/s11845-019-02107-3

[28] Foulkes RE , Heard G, Boyce T, Skyrme R, Holland PA, Gateley CA. Duct excision is still necessary to rule out breast cancer in patients presenting with spontaneous bloodstained nipple discharge. Int J Breast Cancer 2011;2011:495315 22295227 10.4061/2011/495315 PMC3262583

[29] Gui G , Agusti A, Twelves D, Tang S, Kabir M, Montgomery C et al INTEND II randomized clinical trial of intraoperative duct endoscopy in pathological nipple discharge. Br J Surg 2018;105:1583–1590 30238438 10.1002/bjs.10990

[30] Hahn M , Fehm T, Solomayer EF, Siegmann KC, Hengstmann AS, Wallwiener D et al Selective microdochectomy after ductoscopic wire marking in women with pathological nipple discharge. BMC Cancer 2009;9:151 19445720 10.1186/1471-2407-9-151 PMC2689244

[31] Lustig DB , Warburton R, Dingee CK, Kuusk U, Pao JS, McKevitt EC. Is microductectomy still necessary to diagnose breast cancer: a 10-year study on the effectiveness of duct excision and galactography. Breast Cancer Res Treat 2019;174:703–709 30607630 10.1007/s10549-018-05109-4

[32] Ohlinger R , Flieger C, Hahndorf W, Paepke S, Blohmer JU, Grunwald S et al Correlation of ductoscopic and histopathological findings and their relevance as predictors for malignancy: a German multicenter study. Anticancer Res 2020;40:2185–2190 32234913 10.21873/anticanres.14179

[33] Richards T , Hunt A, Courtney S, Umeh H. Nipple discharge: a sign of breast cancer? Ann R Coll Surg Engl 2007;89:124–126 17346403 10.1308/003588407X155491 PMC1964556

[34] Simpson JS , Connolly EM, Leong WL, Escallon J, McCready D, Reedijk M et al Mammary ductoscopy in the evaluation and treatment of pathologic nipple discharge: a Canadian experience. Can J Surg 2009;52:E245–E248 20011159 PMC2792391

[35] Wong Chung JERE , Jeuriens-Van De Ven SAH, Van Helmond N, Wauters CAP, Duijm LEM, Strobbe LJA. Does nipple discharge color predict (pre-) malignant breast pathology? Breast J 2016;22:202–208 26799061 10.1111/tbj.12544

[36] Simmons R , Adamovich T, Brennan M, Christos P, Schultz M, Eisen C et al Nonsurgical evaluation of pathologic nipple discharge. Ann Surg Oncol 2003;10:113–116 12620904 10.1245/aso.2003.03.089

[37] Gray RJ , Pockaj BA, Karstaedt PJ. Navigating murky waters: a modern treatment algorithm for nipple discharge. Am J Surg 2007;194:850–855 18005783 10.1016/j.amjsurg.2007.08.027

[38] Mathis KL , Hoskin TL, Boughey JC, Crownhart BS, Brandt KR, Vachon CM et al Palpable presentation of breast cancer persists in the era of screening mammography. J Am Coll Surg 2010;210:314–318 20193894 10.1016/j.jamcollsurg.2009.12.003

[39] Moy L , Heller SL, Bailey L, D’Orsi C, DiFlorio RM, Green ED et al ACR Appropriateness Criteria^®^ palpable breast masses. J Am Coll Radiol 2017;14:S203–S224 28473077 10.1016/j.jacr.2017.02.033

[40] Balci FL , Feldman SM. Interventional ductoscopy for pathological nipple discharge. Ann Surg Oncol 2013;20:3352–3354 23975311 10.1245/s10434-013-3181-5

[41] Mansel R , Webster D, Sweetland H. *Benign Disorders and Diseases of the Breast*. 2009. http://www.lavoisier.fr/notice/fr283071.html (accessed 1 June 2022)

[42] Chang YK , Chen CTH, Wang M, Yang Y, Mark B, Zheng AQ et al Could ductoscopy alleviate the need of microdochectomy in pathological nipple discharge? Breast Cancer 2020;27:607–612 32008216 10.1007/s12282-020-01051-w

